# Subjective touch sensitivity leads to behavioral shifts in oral food texture sensitivity and awareness

**DOI:** 10.1038/s41598-021-99575-4

**Published:** 2021-10-12

**Authors:** R. Pellegrino, C. McNelly, C. R. Luckett

**Affiliations:** Department of Food Science, University of Tennessee, Knoxville, TN USA

**Keywords:** Psychology, Human behaviour

## Abstract

Neurotypical individuals have subjective sensitivity differences that may overlap with more heavily studied clinical populations. However, it is not known whether these subjective differences in sensory sensitivity are modality specific, or lead to behavioral shifts. In our experiment, we measured the oral touch sensitivity and food texture awareness differences in two neurotypical groups having either a high or low subjective sensitivity in touch modality. To measure oral touch sensitivity, individuals performed discrimination tasks across three types of stimuli (liquid, semisolid, and solid). Next, they performed two sorting exercises for two texture-centric food products: cookies and crackers. The stimuli that required low oral processing (liquid) were discriminated at higher rates by participants with high subjective sensitivity. Additionally, discrimination strategies between several foods in the same product space were different across the groups, and each group used attributes other than food texture as differentiating characteristics. The results show subjective touch sensitivity influences behavior (sensitivity and awareness). However, we show that the relationship between subjective touch sensitivity and behavior generalizes beyond just touch to other sensory modalities.

## Introduction

Humans vary in physiological functioning, including our response to sensory stimuli. Differences in physiological functioning have long been documented under the umbrella term *individual response stereotypy* and include somatic, autonomic, and brain differences^1–5^. Differences in response may come in the form of sensitivities to a specific sensory stimulus or generalized to a broader range of experiences and may refer to hypersensitivity (i.e., over-responsiveness) or hyposensitivity (i.e., under-responsiveness). Atypical sensory processing has been associated with several clinical conditions such as Tourette’s^6^, migraines^7^, and autism spectrum disorder, ASD^8^, but may also affect healthy adults to a lesser extent^9–11^.

There are several distinctions between different sensory sensitivities within atypical sensory processing: behavioral sensory sensitivity, neural sensory sensitivity, and subjective sensory sensitivity. Subjective sensory sensitivities are self-reported individual differences reported through questionnaires, and accounts for most of the studies done on atypical sensory processing. Behavioral sensory sensitivities, on the other hand, are individual differences in the ability to discriminate or detect sensory stimuli, while neural sensory sensitivities are excitation differences among receptors and/or across neural networks to a given stimulus. Currently, there is an open debate on how these measures of sensitivity relate to each other and whether or not they rely on comparable basic mechanisms^12^.

Recently a mathematical representation based on physiological evidence has been proposed to describe a range of possible relationships between subjective, neural, and behavioral sensitivity^12^. In general, this mathematical model sums the signal and noise for which more signal leads to better behavioral outcomes such as discrimination. However, this framework has been rooted in atypical populations, rather than sensitivity differences in neurotypical individuals. Additionally, this framework is focused on general sensory sensitivities rather than modality-specific ones. Modality-specific behavioral sensitives’ have been shown in both clinical^13–15^ and non-clinical groups^9,16–19^, but the relationship of these behavioral sensitivities with specific subjective sensitivity is less clear^12^. Multiple sensory modalities are typically reported to be affected on questionnaire measures of atypical sensory sensitivity^20,21^ which suggests a central origin (i.e., brain-level) rather than a peripheral origin (e.g., at the level of receptors or ascending nerve fibers).

In the context of food, studies have also not considered subjective touch sensitivities may be finely tuned to a specific behavioral outcome such as texture perception. An increase in subjective sensitivity has been associated with increased picky eating and less food intake in children with reports measuring general sensory by averaging across modalities^22–24^. Similarly, behavioral reports, through tactile appraisal, have shown children and adults with higher hand touch sensitivities show more selectivity with food^17,18^. These studies clearly show that subjective and behavioral sensory sensitives can lead to changes in liking of food. Previously we showed that subjective touch sensitivity increased motivations to reject a food and these motivations may not be specific to the sensation of touch, but generalize to several sensations experienced during eating such as smell and taste^25^. However, our conclusions were based on subjective behavioral reports of rejection. An open question from previous work was whether this ‘generalized sensory sensitivity’ leads to specific or non-specific behavioral shifts.

Behavioral touch sensitivity differences exist within a neurotypical population^26,27^ and across the human body with the tongue being more sensitive than the finger^28–30^. Individuals with high lingual touch sensitivity (measured with Von Frey Hairs on the tongue) could discriminate between the grittiness of chocolates better than those with low sensitivities^31^. Similarly, those sensitive to roughness on the tongue show higher sensitivity to some astringent compounds (e.g., epigallocatechin gallate), but not others (tannic acid)^32^. However, other studies have shown no effect, in which participants with high oral tactile sensitivity were not more able to discriminate gummy stimuli of hardness than participants in the bottom quartile of oral tactile sensitivity^19^. The conflicting nature of the reports on the relationship between laboratory measures of behavioral oral tactile sensitivity and food texture perception are likely due to how food texture sensitivity was measured. For example, the two studies which found a link between food texture perception and laboratory measures implemented specified discrimination tasks which focus on single attributes. Conversely, no relationship was found between oral tactile sensitivity and food texture discrimination when using an unspecified discrimination task, suggesting an attentional component to food texture discrimination^19^.

This study set out to explore the specific sensitivities related to touch in the context of food in two ways. Firstly, to address the relationship between subjective and behavioral oral touch sensitivities in a neurotypical cohort, we placed individuals into two groups depending on their subjective touch sensitivity, either high or low. These individuals then underwent several oral texture discrimination tasks. Secondly, modality awareness while eating was explored by having each group rank several products in the same food domain space. In several atypical processing models using a signal detection framework^33,34^ a decrease in behavioral outcome accompanies an increase in subjective sensitivity due to an increase in noise. This notion formed the basis of our hypothesis. As neurotypical individuals often span into atypical processing domains^9,10,21^, we expect a decrease in behavioral outcomes with increased subjective touch sensitivity and for this relationship to be generalized across other modalities involved in eating (e.g. appearance, flavor) rather than specific to touch (texture).

## Materials and methods

### Participants

One-hundred and forty-three individuals started this study by completing the touch sensitivity subscale of the Sensory Perception Quotient (SPQ). From that group fifty-seven healthy, neurotypical individuals were selected from the 25th and 75th quartile scores to make up two groups with high [n = 29 (19 females, age ± sd = 36.1 ± 13.8 years)] and low [n = 28 (22 females, age ± sd = 41.4 ± 13.7 years)] touch sensitivity.

All individuals reported no sensory impairments nor dietary restrictions, and there were no significant differences between groups for age (*p* = 0.15) or sex (*p* = 0.28). Additionally, all participants were screened for liking sweet confectionaries. Participants provided written informed consent prior to beginning study and were compensated upon completion of the study. This experiment was conducted in compliance with the Declaration of Helsinki for studies on human subjects and approved by the University of Tennessee IRB review for research involving human subjects (IRB# 20-05876-XM).

### Subjective touch sensitivity

Individuals were separated into high and low touch sensitivity groups prior to participating in the main part of the study using the touch sensitivity subscale of the Sensory Perception Quotient (SPQ). The SPQ was developed for adults with and without autism^21^ and the touch sensitivity subscale correlates with sensory-affective motives and behaviors related to food rejection in neurotypical adults^25^.

### Texture discrimination

Three stimuli were used to test the ability of participants to discriminate foods by their texture, each representing a different type of food: solid (gummy), semisolid (icing), and liquid (fruit-flavored beverage). Each stimulus had two variants, in which one component of the recipe was modified to change the texture. The hardness of the gummy was modified by the bloom strength of the gelatin. The amount of white, flavorless, nonpareils in a white icing were used to modify the particle texture, and xanthan gum was used to increase the viscosity of the fruit-flavored beverage. The formulation of the stimuli were optimized to ensure the discrimination task could be completed above chance (> 33%, see below for details), but was not too easy to guard against a ceiling effect (< 66%). The stimuli were optimized in preliminary studies (see supplementary for more details). All stimuli formulations can be found in the supplementary materials (Supp. Table S1).

The discrimination task chosen for this study was the triangle test, where individuals are given three samples of a stimulus, two of them are identical and one is different. In this task, the participants are asked to identify the odd sample, and an answer is recorded as correct/incorrect. This discrimination task was unspecified, meaning the participants were not informed on what attribute to look for when attempting to differentiate between the foods. After making their selection, the participants were asked about their confidence in their choice as well as what sensory cue helped them decipher the difference. The arrangement of the samples and the target (odd sample) was randomly determined per individual. Each stimulus was done in triplicate; therefore, a total of 9 discrimination tests were done across the sessions.

### Texture awareness

A modified flash profiling procedure was used to examine the texture awareness of the participants. Five products from 2 different product types (chocolate chip cookies and grain-based crackers) were tested (Supp. Table S2). For each product type, individuals were given 5 different products and were instructed to examine/try each product while thinking of defining sensory characteristics. They were then asked to reexamine the products and write down sensory terms that differentiated products from each other while avoiding evaluative terms (e.g. yummy, gross). They were encouraged to try the products as much as they needed and were given an ample amount of each. Next, they were given 32 terms as a check-all-that-apply (CATA) and asked to check which one they had on their list. The term types were the same for both product spaces (37.5% flavor, 34.5% texture, 9.4% taste, 9.4% appearance, 6.2% chemesthetic, and 3.0% sound), but had different terms (Table 1) compiled from published lexicons^35^. For each term selected, individuals were asked to rank the products from most to least in relation to that term. If there were terms not in the CATA, they were asked to list them in a provided space.

**Table 1:** List of term types and associated terms for products profiled.

Term type	Terms	
	Chocolate chip cookie	Cracker
Flavor	Raw grain	Raw grain
Flavor	Toasted	Toasted
Flavor	Chocolate	Hay-like/grassy
Flavor	Cocoa	Caramelized
Flavor	Buttery	Buttery
Flavor	Cooked Milk	Herb/Spice
Flavor	Vanilla	Burnt
Flavor	Caramel	Earthy/Green
Flavor	Brown sugar/molasses	Cardboard
Flavor	Nutty	Nutty
Flavor	Heated Oil (Rancid)	Heated Oil (Rancid)
Flavor	Baking Soda	Baking Soda
Chemesthesis	Astringent	Astringent
Chemesthesis	Spicy / Heat	Spicy / Heat
Taste	Sweet	Sweet
Taste	Salty	Salty
Taste	Bitter	Bitter
Texture	Rough	Rough
Texture	Crumbly	Crumbly
Texture	Dense	Dense
Texture	Cohesive	Dry
Texture	Crispy	Crispy
Texture	Crunchy	Crunchy
Texture	Moist	Moist
Texture	Soft	Grainy / Gritty
Texture	Hard	Hard
Texture	Oily/greasy	Oily/greasy
Texture	Adhesive/sticky	Abrasive / Sharp
Appearance	Chip Count	Lumpy Look
Appearance	Toasted Look	Toasted Look
Appearance	Irregular Form	Irregular Form
Sound	Crackle Sound	Crackle Sound

### Procedure

The test was completed across three sessions separated by 24 h. For each session, individuals first completed discrimination tests (in triplicate) and then modified flash profiling. On the third day there was no product space to profile after the discrimination task.

### Statistical analysis

All analysis was done in R (3.0.4), all code and data can be found here: https://osf.io/d4avz/. For the discrimination tests, the R package sensR^36^ was used to calculate d′ for each sensitivity group with each stimulus and these were compared using three, two-tailed z-tests, in which the d′ values from each group were compared for each stimuli set separately. p-values were calculated using the *pnorm* function. Additionally, ratings of confidence were analyzed with a two-way ANOVA (stimulus, group, and their interaction) while the sensory cue was plotted for visual interpretation.

Texture awareness data was analyzed in two ways. First, each selected attribute was counted within its respective sensory category (flavor, texture, taste, appearance, chemesthetic, and sound). If participants added extra terms outside of the CATA questions, they were added to the respectable count. A mixed model fit with a Poisson distribution using the R package lmer^37^ was performed using sensory category as a within-subject variable and sensitivity group as the between-variable along with their interaction. Subject and product space were set as a random variable. Next, the CATA terms/categories and their respective rankings of products within the same domain were incorporated into a multiple factor analysis (MFA) model using the FactoMineR package^38^. Sensitivity and products were set at categorical variables within this space and their variance of the model was compared. Thus, two different MFAs were done, one for each product space (i.e., cookies and crackers). For each MFA, terms that were chosen less than ~ 20% for both groups were excluded from the analysis.

## Results

**Figure 1:**
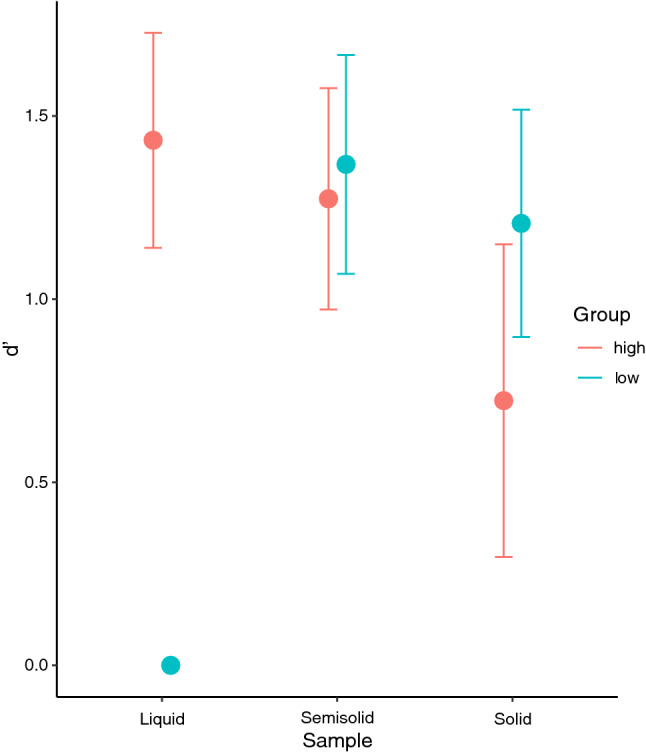
Discriminatory differences between touch sensitivity groups measured with d-prime (d′). Both groups discriminated solid and semisolid samples at comparable rates, but the high sensitivity was notably better at discriminating the liquid stimuli than the low sensitivity group.

Using signal detection theory d′ values were calculated for each stimulus. d′ values estimate the discriminability between two stimuli, where a d′ of zero represents identical samples and a d′ greater than 1 signifies the detection threshold for a population. As shown in Fig. 1, when looking at the liquid stimuli, a direct comparison between the high sensitivity group (d′ = 1.434) and the low sensitivity group (d′ = 0) is not possible in this case because variance of d′ cannot be estimated when d′ = 0. However, a one-sided exact binomial test comparing the d′ of the high sensitivity group to 0 reveals the d′ of the high sensitivity group to significantly greater than zero (p = 0.002). Regarding the solid stimuli, the high sensitivity group (d′ = 0.723) was not significantly different than low sensitivity group in their discriminatory ability (d′ = 1.207, p = 0.359). The results were similar for the semisolid stimuli, where the high sensitivity group (d′ = 1.274) was not significantly different than low sensitivity group in their discriminatory ability (d′ = 1.368, p = 0.825).

**Figure 2:**
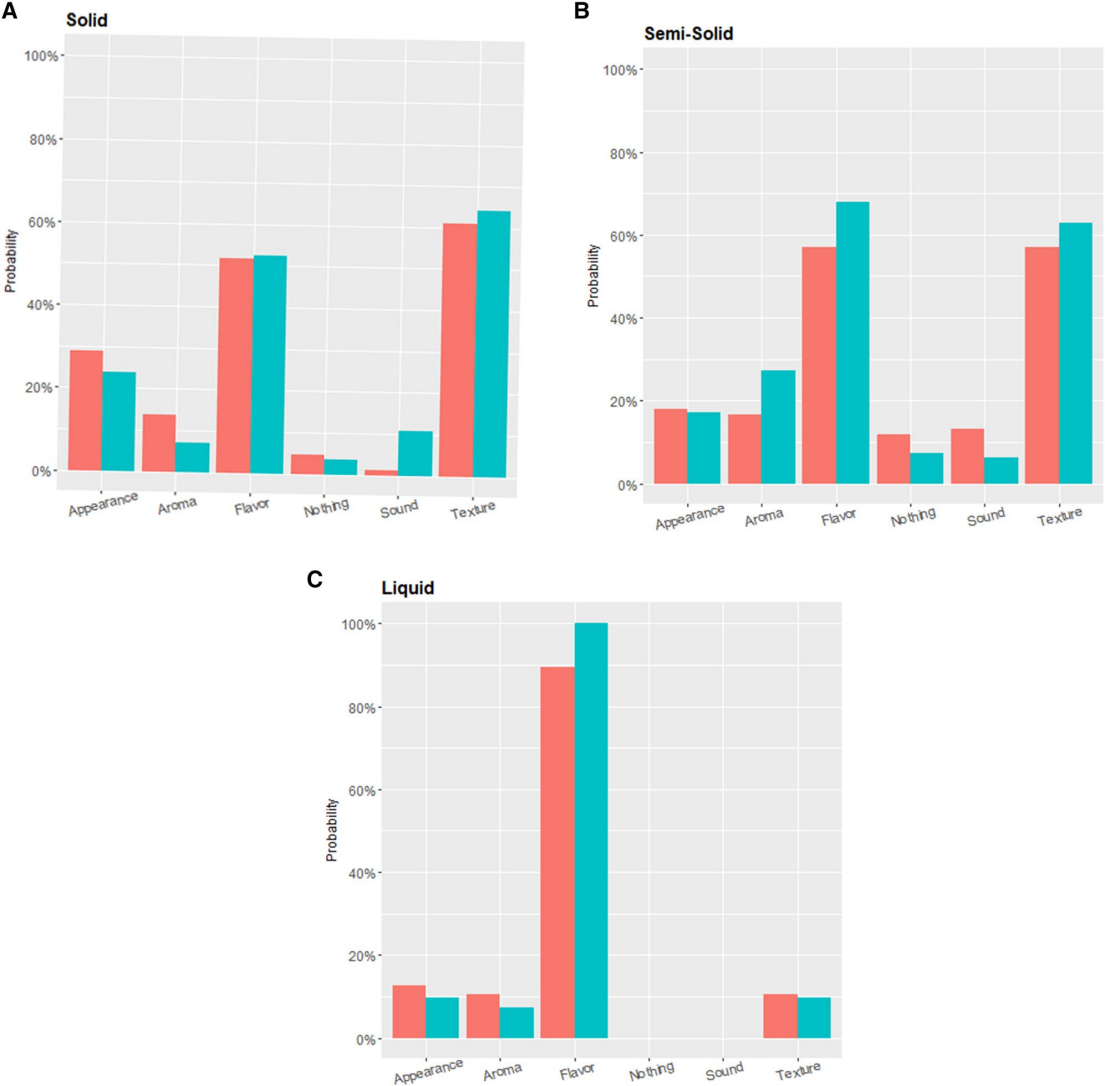
Distribution of sensory cue used for discrimination between touch sensitivity groups. (**A**) The solid stimulus (gummy) as well as the semisolid (icing, **B**) had a scattered distribution of sensory cues revealing a similar complexity for the discrimination tasks. (**C**) The liquid stimulus (Kool-Aid) shows a relatively simple stimulus with flavor being the main sensory cue used for discrimination.

The confidence ratings showed an interaction (F_2,280_ = 3.16, *p* = 0.04), where high touch sensitivity individuals had more confidence in the liquid stimulus task than low sensitivity (p < 0.001). Similarly, the liquid stimuli elicited a clear singular sensory signal, flavor (Fig. 2C). However, sensory alone does explain the other two stimulus results as both were complex (Fig. 2A,B) thus the additional mechanical involvement may be leading to the discrimination discrepancy between the two groups.

**Figure 3:**
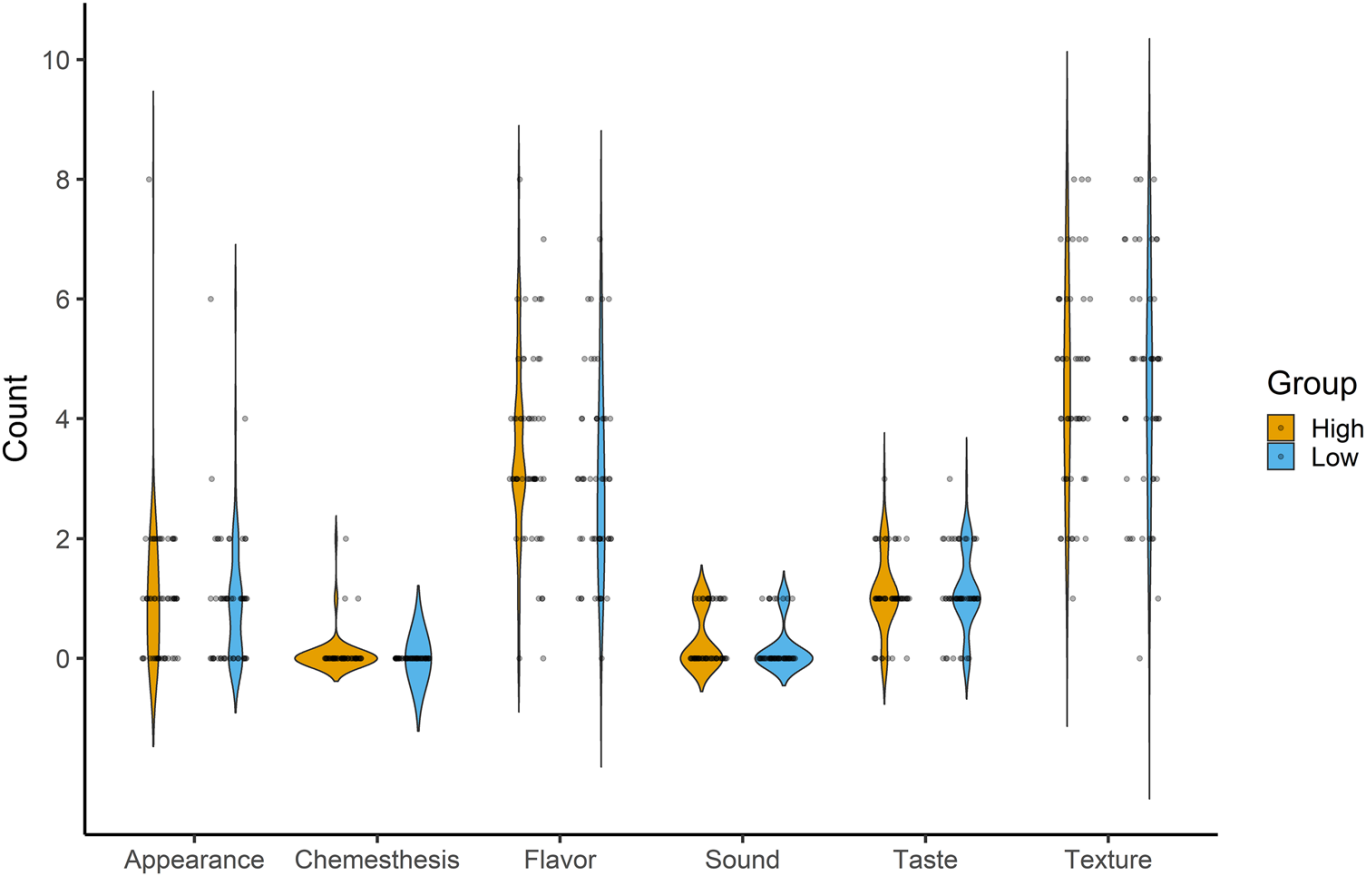
Violin plot with usage frequency of different term types among high and low touch sensitivity groups. No differences were seen across the sensitivity groups. Texture terms were chosen the most followed by flavor and taste/appearance. Terms include those presented in the CATA term bank as well as those defined by the individual.

There were no differences in the type of terms used to profile the products (F_5,658_ = 0.66, p > 0.05; Fig. 3). Both groups used more texture terms followed by flavor and then taste/appearance. Chemesthesis and sound terms were used the least.

**Figure 4:**
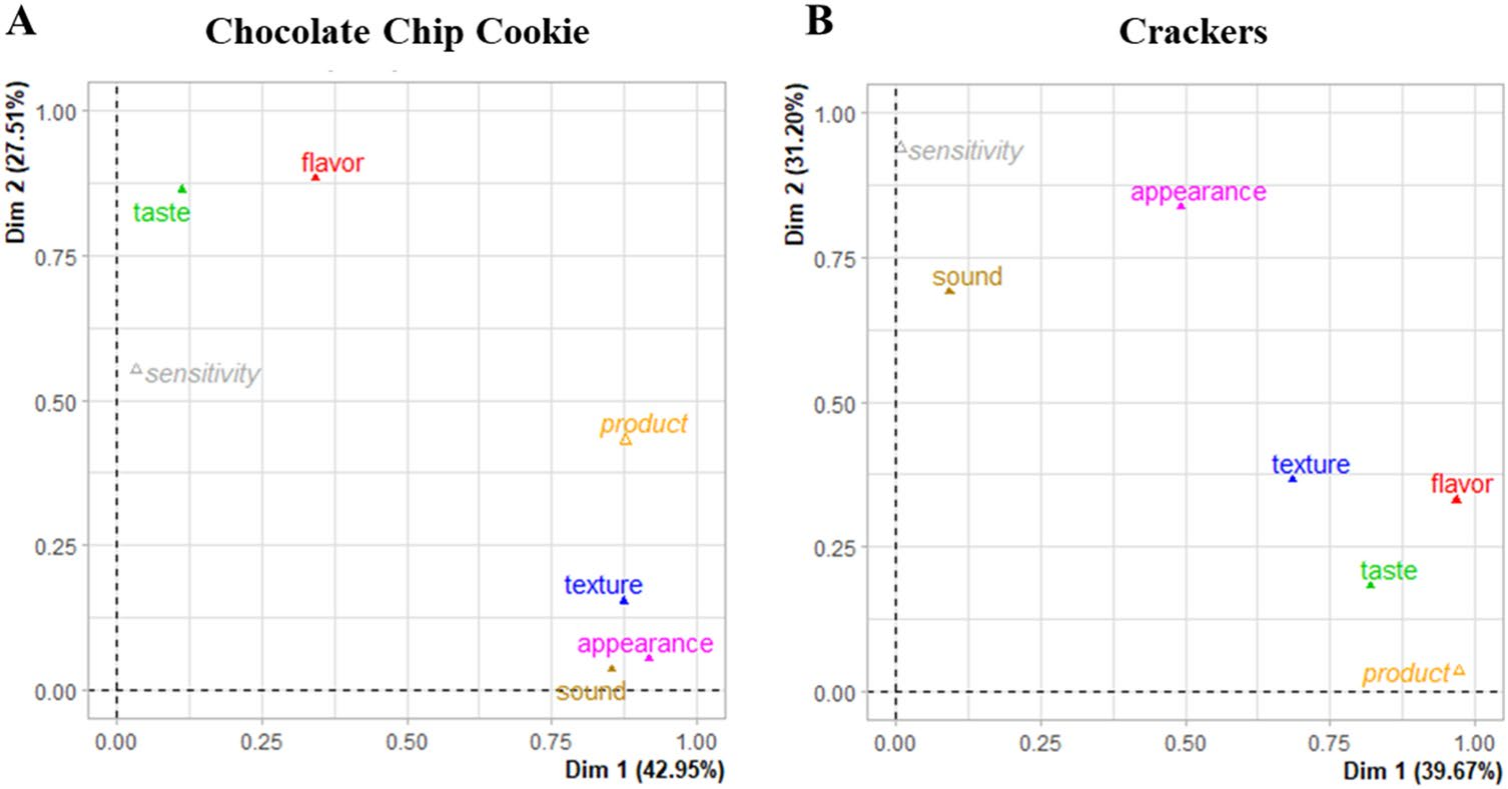
Weighting of sensory term types by sensitivity group and products by products. Two dimensions describe 70% of the space for each product in which the first dimension describes agreement in term types between groups of touch sensitivity and the second-dimension disagreement in term types. (**A**) and (**B**) shows the terms differ among groups, but they are dependent on the food and are not specific to texture.

**Figure 5:**
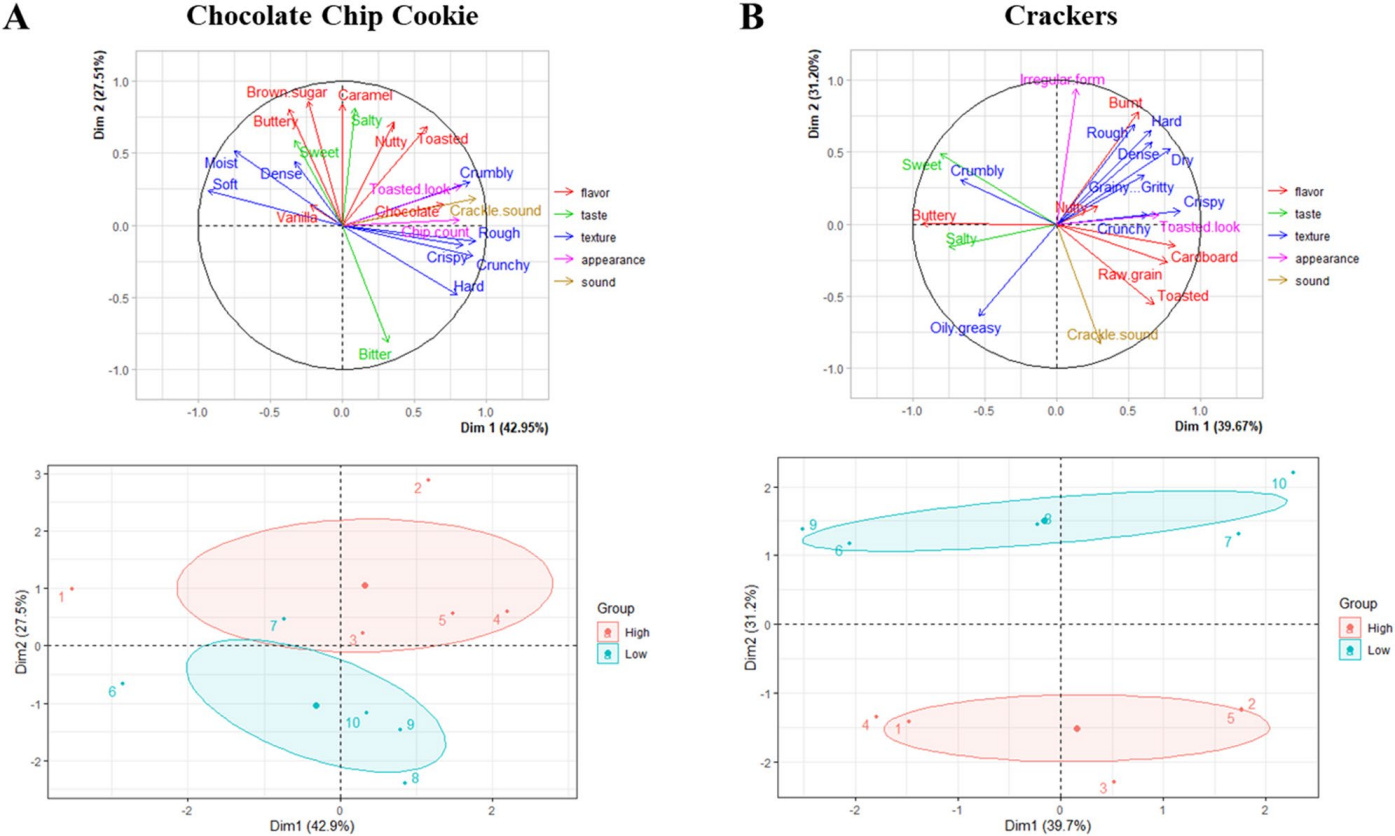
Multiple Factor Analysis plot showing individual term ranks between groups by product. High and low touch sensitivity groups used different terms to rank the product space, but differences were dependent on the product space being profiled (**A**, chocolate chip cookies, and **B**, crackers).

Although the frequency of term usage was not different, the way terms were ranked within the product space was different between sensitivity groups. For each product, 70% of the variance was explained by the first two dimensions with one distinguishing between products and the other groups (Fig. 4). Group differences were not specific to touch but generalized across different senses depending on the food type. Term ratings on the second dimension were different between sensitivity groups for both products, cookies (p = 0.01) and crackers (p < 0.001). For chocolate chip cookies (Fig. 5A), the high sensitivity group gave more weight to several flavor terms [brown sugar (*r* = 0.86, *p* = 0.001), caramel (*r* = 0.84, *p* = 0.002), buttery (*r* = 0.81, *p* = 0.004), nutty (*r* = 0.72, *p* = 0.02), and toasted (*r* = 0.68, *p* = 0.03)] and salty taste (*r* = 0.81, *p* = 0.004) while low sensitivity gave higher importance to bitter taste (*r* = − 0.81, *p* = 0.004). No texture terms were differently rated across the group (p > 0.05). Different term usage appeared with another product space, crackers (Fig. 5B). For crackers, the high sensitivity group gave more weight to sound [crackle sound (*r* = − 0.83, *p* = 0.003)] and one texture [oily/greasy (*r* = − 0.64, *p* = 0.046)] while low sensitivity gave higher importance to an appearance term [irregular form (*r* = 0.94, *p* < 0.001)], a flavor term [burnt (*r* = 0.77, *p* = 0.008)], and two texture terms [rough (*r* = 0.69, *p* = 0.03) and hard (*r* = 0.65, *p* = 0.05)].

## Discussion

In this study, we show that subjective touch sensitivity differences exist within a non-clinical population and have behavioral implications. Similar to our past study^25^, we show subjective touch sensitivity generalizes to other senses involved in eating (e.g. appearance, flavor) rather than being specific to touch related attributes (food texture). The type of behavioral sensitivity and awareness within the mouth is also food dependent. We will discuss these findings in terms of signal processing of sensory inputs and relate it to the current research of sensory processing in atypical populations.

### Behavioral sensitivity

There are several distinctions between different sensory sensitivities within sensory processing: behavioral sensory sensitivity, neural sensory sensitivity, and subjective sensory sensitivity. A signal detection framework, based on atypical processing studies, has been used to describe a range of possible relationships between subjective, neural, and behavioral sensitivity which balances signal and noise^12^$.$In several atypical processing models^33,34^, this framework leads to a decrease in behavioral outcome with an increase in subjective sensitivity due to an increase in noise. However, in our study, we show that signal and noise may shift depending on the food. For solid and semi-solid foods there were no differences in discriminatory ability among subjective touch sensitivity groups; however, individuals with high touch sensitivity were able to detect and use a single sensory signal for the liquid stimuli, with confidence, leading to an improvement in discrimination. Liquid stimuli have a quick oral transit time as they are a relatively simple stimuli and do not require much oral processing to consume^39^. Indeed, individuals have been found to be able to detect tactile differences at miniscule changes in viscosity in liquid stimuli^40^. The difference between our findings across the food products used could be food dependent, based on oral processing and transit time as mentioned earlier or due to more general task-dependent factors. For example, a recent study showed neurotypical individuals with higher sensitivity had a narrow dynamic range of vibrotactile response, but this relationship did not exist for tactile threshold^9^.

The food-dependency of signal detection in our study suggests that the noise depends on the signal following Excitation-to-Inhibition Ratio models of atypical processing^33^. Yet, to fully understand the relationship between subjective and behavioral sensory processing a neural component is needed.

### Generalization across modalities

A food-dependency was also shown in sensory awareness when distinguishing different products in the same space. Here, high and low touch sensitivity individuals used different sensory strategies for each food, and these were not specific to texture. Similar to our past study^25^, subjective touch sensitivity generalizes to other senses in food perception. In atypical populations, this generalization is commonly observed^20,21^ as traits are considered innate and not learned through perceptual experience^41–44^. For instance, a large twin study showed that among 12,419 dyads 66–71% of sensory reactivity was heritable^44^. So, does the abnormal signal processing happen for all sensory inputs thus measuring one can be a proxy for the others? There are not many studies directly testing this hypothesis and there is evidence of sensory specificity in some disorders like misophonia^45^ or photosensitive epilepsy^46^. Even in our study, we are assuming heightened awareness of different sensory inputs other than texture between touch-sensitive groups demonstrates generalization. This is an outstanding question in theoretical accounts of individuals differences in sensory sensitivity that future studies should attempt to address. The need for neural mechanisms will again need to be incorporated into the study design as the central origin (i.e., brain-level) rather than the peripheral origin (e.g., at the level of receptors or ascending nerve fibers) must be determined. Additionally, we are making a comparison between mostly autism research with our neurotypical population, as the former has been comprehensibly studied, but other motives to sensitivity differences may be present. For instance, the Intense World Theory claims that hypersensitivity is cooccurring with increased emotions or moods (e.g., anxiety)^47^. Similarly, other exogenous and endogenous factors most likely affect these relationships such as attention through prioritization and stimuli salience^48^.

### Future directions/limitations

This study used food texture discrimination as a measure for behavioral sensitivity. This decision prioritized ecological validity, but a more direct measure of tactile sensitivity such as point pressure sensitivity^31^, bite force sensitivity^49^, and/or roughness sensitivity^32^, could be implemented if eating behavior and food choice were not of interest. These assessments of oral tactile sensitivity could have led to different results, as previous research has shown no relationship between oral tactile sensitivity and food discrimination through texture^19^. Furthermore, future directions could also deviate completely from focusing on the oral cavity, and address relationships between subjective and behavioral tactile sensitivity in the hands and/or fingers. One major factor here is sensitivity to touch depends on the body part in which the oral cavity, and specifically the tongue, has been documented to be extremely sensitive to touch when compared to the fingers^28–30^. Because of these differences in normative tactile sensitivity, specific areas of the body could see a different relationship between subjective and behavioral touch sensitivity.

## Conclusion

In two neurotypical groups having either a high or low subjective touch sensitivity showed differences in a series of tasks to assess their oral touch sensitivity and texture awareness of foods. This reflects observations in populations showing autistic-related traits or diagnosis. For example, there is overlap between ASD patients who show atypical processing and the general population^9,10,21^. Yet, we show a benefit to behavioral outcomes related to oral processing for those with higher subjective touch sensitivities. The stimuli that required low oral processing (liquid) were discriminated at higher rates by individuals with high subjective touch sensitivity compared to those with low touch sensitivity. Additionally, discrimination strategies between several foods in the same product space were different across these touch sensitivity groups, and each group used attributes other than texture as differentiating characteristics. The results show subjective touch sensitivity influences behavior (sensitivity and awareness). However, we show that the relationship between subjective touch sensitivity and behavior is not specific to texture, but extends to other modalities involved in eating.

## Supplementary Information

Supplementary Information.
