# Supplementary material for: Subjective touch sensitivity leads to behavioral shifts in oral food texture sensitivity and awareness
Source: Sci Rep. 2021 Oct 12;11:20237. doi: 10.1038/s41598-021-99575-4 (PMC8511070; doi:10.1038/s41598-021-99575-4)
Supplement: Supplementary file 1 — Supplementary Information. [file 41598_2021_99575_MOESM1_ESM.docx]

**Supplementary Information for**

Subjective Touch Sensitivity Leads to Behavioral Shifts in Food Texture Sensitivity and Awareness

Robert Pellegrino, Chloe McNelly, and Curtis R. Luckett

Curtis R. Luckett

Email: [cluckett@utk.edu](mailto:cluckett@utk.edu)

**This PDF file includes:**

S1: Stimulus design

- Table S1 to S1

S2: Product profiles

- Table S1 to S2

**Other supplementary materials for this manuscript include the following:**

All data, processing code and analysis scripts are available in an online OSF repository <https://osf.io/d4avz/>

S1: Stimulus design

Stimuli were designed in a preliminary study. The formulation of the stimuli were optimized to ensure the discrimination task could be completed above chance (>33%, see below for details), but was not too easy to guard against a ceiling effect (< 66 %). This was achieved by running triangle tests, similar to those ran in the main study, across several subjects (N=10) in triplicate in which variant ingredient was modified.

Table S1. Stimulus formulas used in texture discrimination.

| **Stimulus** | **Ingredients** | **Amount (%)** | **Variant** |
| --- | --- | --- | --- |
| Solid (Gummy) | Distilled water | 22.75% |  |
|  | 200 bloom gelatin | 8.75% | 190 bloom |
|  | Sucrose | 22.00% |  |
|  | Sorbitol | 2.20% |  |
|  | Citric Acid | 0.30% |  |
|  | Glucose syrup | 44.00% |  |
|  | Red dye | 0.25% |  |
|  | Strawberry extract | 0.15% |  |
| Semi-solid (Icing) | White icing | 100.00% |  |
|  | White nonpareils | 8.00% | 12.00% |
| Liquid | Distilled water | 91.00% |  |
| (Fruit-flavored beverage) | Koolade Tropical Punch | 0.22% |  |
|  | Sucrose | 9.00% |  |
|  | Xanthan gum | 0.00% | 0.05% |

S2: Products profiled

Table S1. Product types and brands used in texture awareness profiling

| **Product** | **Brand** |
| --- | --- |
| Chocolate Chip Cookies | Keebler Soft Batch Chocolate Chip Cookies |
|  | Chips Ahoy! Original Chocolate Chips Cookies |
|  | Kroger ChipMates Original Chocolate Chip Cookies |
|  | Pepperidge Farm Thin & Crispy Milk Chocolate Chip Cookies |
|  | Crumbdillyicious Chocolate Chip Cookies |
| Crackers | Ritz Original Crackers |
|  | Breton Original Crackers |
|  | Premium Original Saltines |
|  | Keebler Original Club Crackers |
|  | Carr's Table Water Crackers |
